# Cognitive Rehabilitation in Multiple Sclerosis in the Period from 2013 and 2021: A Narrative Review

**DOI:** 10.3390/brainsci12010055

**Published:** 2021-12-30

**Authors:** Bruno Brochet

**Affiliations:** Neurocentre Magendie, INSERM U 1215, Université de Bordeaux, 33077 Bordeaux, France; bruno.brochet@u-bordeaux.fr

**Keywords:** multiple sclerosis, cognitive rehabilitation, cognition, memory, review

## Abstract

Background: In recent years, several blinded randomized controlled trials (RCT) have been conducted on cognitive rehabilitation (CR) in adults with multiple sclerosis (MS). Objective: To review all blinded RCTs on CR in MS published since 2013. Methods: The National Library of Medicine database (Medline) and PSYCINFO were searched using the keywords MS and CR or cognitive training or NP rehabilitation or memory rehabilitation or attention rehabilitation. Results: After the exclusion of some papers not specifically focused on CR, a final list of 26 studies was established. The papers belong to three main categories: individual specific rehabilitation (8studies), group rehabilitation (4 studies), and computerized training (CT) (14 studies), while one study combined group rehabilitation and CT. Among the individual rehabilitation studies, 5 were devoted to memory, and most of the 19 other selected studies were about several cognitive domains. Most of the studies mainly concerned RRMS patients, except for 2 studies that were carried out exclusively in progressive forms. Despite the methodological limitations of some studies and the great heterogeneity of the protocols, the results are generally in favor of the efficacy of CR in neuropsychological tests. Conclusion: Recent blinded RCTs about CR in MS show promising results.

## 1. Introduction

Treating cognitive impairment (CI) in multiple sclerosis (MS), the leading cause of disability due to nontraumatic neurological disease in young adults, is an important challenge [1]. The contribution of CI to disability in MS has been increasingly recognized, and CI has been shown to decrease health-related quality of life (HR-QoL), even in the early stages of the disease [2]. CI negatively affects daily activities such as driving, vocational status, absenteeism, and instrumental activities in persons living with MS [3]. No medication has proven to have a consistent symptomatic effect on CI in MS, and disease-modifying therapies only have a limited impact on CI progression [4].

CI in MS is dominated by a slowdown in information processing speed (IPS), as well as by disturbances of more specific cognitive functions such as attention, episodic memory (EM), working memory (WM), and executive function (EF) [5]. If a relatively circumscribed alteration in IPS linked to a specific process deficit can occur, changes in IPS can alter other cognitive processes and usually reflect cognitive functioning and efficiency. The alteration of IPS has consequences for WM, attention, EF, and EM. IPS impairment predicts later disability vocational status and changes in quality of life [3].

Cognitive rehabilitation (CR) is the most promising approach for treating MS-related CI, despite important methodological shortcomings. CR programs could include techniques designed to improve specific domains of cognitive function such as memory, attention, or executive functions, but they can also include psychotherapy targeting emotional symptoms, behavioral interventions, and interventions targeting psychomotor issues such as motor–cognitive interference. In this review, we will focus only in interventions designed to improve specific cognitive domains in MS.

A wide variety of neuropsychological tests have been used in the assessment of cognitive impairment in MS. A panel of experts recently proposed recommendations [3] for screening tests—the Symbol Digit Modalities Test (SDMT) [6], the Processing Speed Test (PST) [7], and the Computerized Speed Cognitive Test (CSCT) [8], a self-report scale (Multiple Sclerosis Neuropsychological Screening Questionnaire (MSNQ) [9]—and recommended brief cognitive batteries: the Brief International Cognitive Assessment for Multiple Sclerosis (BICAMS) [10], the Brief Repeatable Neuropsychological Battery (BRNB) [11], and the Minimal Assessment of Cognitive Function in MS (MACFIMS) [12]. While most of the CR studies used the SDMT, the other assessment tests varied widely from study to study.

In 2013, Amato et al. reviewed the available studies at that time on CR and listed 23 studies [1]. However, methodological limitations in many studies have led to disappointing results, and well-designed studies are still scarce, as shown by another contemporary review [13]. Many studies lack a randomized controlled design that includes passive or active control conditions, primary neuropsychological (NP) endpoints identified a priori, evidence of the sustainability of CR, and the inclusion of near and far transfer outcomes. Tertiary outcomes of QoL, metacognition, or other patient-reported outcomes (PROs) have rarely been used. Since this time, many studies have been performed, and many were randomized controlled trials (RCT). We review here the RCTs with blinded assessments that have been published between 2013 and 2021.

## 2. Methods

The National Library of Medicine database (Medline) was searched at https://pubmed.ncbi.nlm.nih.gov/ using the following keywords on 18 November 2021.

MS and CR or cognitive training or NP rehabilitation or memory rehabilitation or attention rehabilitation.

Inclusion criteria were as follows: RCTs, published in English as full papers, blinded assessment, cognitive intervention (excluding exercise training and cognitive and motor joint intervention such as dual tasks and intervention combined with brain stimulation, music therapy, etc.), specified endpoint focused on cognitive outcomes (NP testing required) (excluding studies focused on fatigue, motor outcome, depression, and anxiety as primary outcomes, feasibility studies, and reports devoted primarily to imaging outcomes). Quantitative results should be presented in the paper, excluding papers about research protocols and qualitative studies. Studies in pediatric-onset MS were not included.

### 2.1. Results

A first search with the terms multiple sclerosis and CR, RCT, 2013–2021, full text resulted in a list of 141 references. A similar search was performed on the PSYCINFO database and resulted in a list of 31 references. After application of the eligibility criteria, 23 papers were selected. After running searches using the other pre-selected keywords (memory rehabilitation and MS), three additional papers were selected. Table 1 presents the list of the 26 studies included in the analysis [14,15,16,17,18,19,20,21,22,23,24,25,26,27,28,29,30,31,32,33,34,35,36,37,38,39]. Papers were classified into three main categories: individual specific rehabilitation (eight studies), group rehabilitation (four studies), and other computerized training (CT) (14 studies), while one study combined group rehabilitation and CT [30]. Most of the studies mainly concern Relapsing–Remitting MS (RRMS) patients, with the exception of two studies carried out exclusively in progressive forms [33,36]. Five studies investigated memory rehabilitation exclusively, while the other studies addressed multiple domains.

### 2.2. Methodological Analysis

All studies were blinded RCTs. Thirteen studies were double blinded and thirteen were single blinded (blinded evaluators) (Table 1). Some bias can be identified. Three studies included patients regardless of the presence of CI [19,24,30]. We did not consider this as an exclusion criterion in our review, but this may have affected the results of these studies. In nine studies, the control group did not have any intervention (waiting list or usual care). However, in the other 17 studies, a placebo or sham intervention was administered in the control group. Several studies were considered pilot studies and thus, their power was limited. The sample size was less than 35 subjects in nine studies and more than 100 subjects in only four studies (Table 1).

**Table 1:** Selected studies.

Study	Blinding	MS Types (%)	CI	n	Cognitive Domains	Type	Setting
Chiaravalloti et al. (2013) [14]	double	RR (64); SP (20), PP (6)	+	86	Memory	Individual	Institution
Cerasa et al. (2013) [15]	double	RR (100)	+	26	Attention	CT	Institution
Rosti-Otajärvi et al. (2013) [16]	single	RR (100)	+	99	Attention	Individual	Institution
Amato et al. (2014) [17]	double	RR (100)	+	88	Attention	CT	Home
De Giglio et al. (2015) [18]	single	RR (100)	+	35	Memory, attention, visuospatial processing and calculations	CT	Home
Hancock et al. (2015) [19]	double	RR (70), SP (17), PP (13)	-	40	IPS and WM	CT	Home
Gich et al. (2015) [20]	single	RR (88), SP (22)	+	43	Multiple	Individual	Institution
Pedullà et al. (2016) [21]	single	RR (61), SP (39)	+	28	WM	CT	Home
Campbell et al. (2016) [22]	double	RR (70), SP (30)	+	35	WM, memory, attention	CT	Home
Charvet et al. (2017) [23]	double	RR (100)	+	135	IPS, attention, WM, EF	CT	TR
Grasso et al. (2017) [24]	single	RR (47), SP (44), PP (9)	-	34	attention, IPS, EF	CT	Institution
Messinis et al. (2017) [25]	single	RR (100)	+	58	Memory, IPS, attention, EF	CT	Institution
Chiaravalloti et al. (2018) [26]	double	RR (100)	+	21	IPS	CT	Institution
Goverover et al. (2018) [27]	double	RR (69), SP (11), PP (20)	+	35	Memory	Individual	Institution
Mani et al. (2018) [28]	single	Not specified	+	34	Memory, attention, EF	Group	Institution
Moussavi et al. (2018) [29]	double	Not specified	+	60	WM	Group	Institution
Stuifbergen et al. (2018) [30]	single	RR (68)	-	183	Memory, attention, problem solving	Group +CT	Institution/Home
Krch et al. (2019) [31]	double	RR (100)	+	20	Memory	Individual	Institution
Brissart et al. (2020) [32]	double	RR (78)	+	110	Memory, EF, language	Group	Institution
Chiaravalloti et al. (2020) [33]	double	SP (57), PP (39)	+	30	Memory	Individual	Institution
Lamargue et al. (2020) [34]	single	RR (83), SP (11), PP (6)	+	35	Attention, IPS, EF, WM	Individual	Real life
Lincoln et al. (2020) [35]	single	RR (65), SP (25), PP (10)	+	449	Attention and memory	Group	Institution
Messinis et al. (2020) [36]	single	SP (100)	+	36	Memory, IPS, attention, EF	CT	Home
Vilou et al. (2020) [37]	single	RR (100)	+	47	Memory, attention, IPS	CT	TR
Chiaravalloti et al. (2021) [38]	double	RR (65), SP (10), PP (15)	+	20	Memory	Individual	Institution
Blair et al. (2022) [39]	single	RR (57), SP (40);PP (3)	+	30	WM	CT	TR

CI: cognitive impairment required in inclusion criteria; CT: computerized training; EF: executive functions; IPS: information processing speed; n = sample size; RR: relapsing–remitting; SP: secondary progressive; PP: primary progressive; TR: telerehabilitation WM: working memory.

Table 2 summarizes the outcomes used in these studies. 

All studies included NP scores as outcomes (which were primary outcomes in 14 studies). Other primary outcomes were used in three studies: perceived deficits in one study [16,40], instrumental activities of daily living (IADL) in one study [30], and HR-Qol in one study [35]. Nine reports did not identify a primary endpoint (Table 3, Table 4 and Table 5).

Most studies incorporated HRQoL measures in their outcomes, but ten did not [15,17,21,23,25,26,28,29,30,37]. Seventeen studies used some self-assessment of cognitive functioning as outcomes, but nine did not [15,19,20,21,25,29,32,36,37].

## 3. Reported Results

Table 3, Table 4 and Table 5 summarized the results of the selected studies. We considered as positive (for primary and secondary outcomes) only a superiority of the group receiving investigational treatment as compared to the control group (with no intervention or placebo/sham intervention) confirmed by ANOVA, ANCOVA, the Mann–Whitney U test, or some equivalent. In some instances, * in the negative results column indicates results that show no difference between the treatment group and the control group, while there was a significant improvement in the treatment group and sometimes in the control group compared to the baseline evaluation.

### 3.1. Individual-Specific Rehabilitation

This category includes five studies on memory rehabilitation [7,20,24,26,31] and three studies on several domains [16,20,34] (Table 3). The rehabilitation was carried out by a research assistant or a trainer in four studies [14,31,32,38], a neuropsychologist in one study [9], speech therapists in one study [34], an occupational therapist in one study [27], and the therapist was not specified in one study [20]. The material used was generally written material, but it could include computer-assisted exercises (three studies) [20,31,34].

#### 3.1.1. Memory

All studies devoted to the rehabilitation of episodic memory came from the same research group of the Kessler Foundation. After an initial pilot study dedicated to the modified-story memory technique (mSMT)-based rehabilitation method [41], which is basically an imagery- and context-based memory retraining program, Chiaravalloti et al. [14] conducted a pivotal study on a larger sample (86 patients) with a positive result on the main criterion, the learning slope of an NP test of EM (California Verbal Learning Test-second edition, CVLT-II), and a positive effect on Functional Assessment of MS (FAMS), an assessment of HR-QoL, as a secondary endpoint. These positive results were maintained at the remote evaluation performed six months after treatment. The second primary endpoint assessing everyday objective memory (RBMT) was also significantly more improved in the treated group than in the control group but with a small effect size. This study did not show any efficacy of booster sessions.

Two other studies were devoted to the m-SMT technique with smaller sample sizes. One study concerned the Spanish adaptation of m-SMT performed in Mexico on 20 patients [31]. The primary endpoint (learning at the Hopkins Verbal Learning Test—Revised, an NP test of EM) was reached, and a secondary outcome about life satisfaction (Satisfaction with Life Scale) was also significantly improved. However, there was no significant improvement in the subjective perception of memory for the Memory Functioning Questionnaire (MFQ). A recent study evaluated the effectiveness of m-SMT in a sample of 30 patients with progressive forms of the disease [26]. The learning curve of the CVLT-II was significantly better in the treatment group than in the control group (primary endpoint), and a significant improvement was also noted on the Frontal Systems Behavior Scale, which is a measure of daily life functioning. However, the second primary endpoint (RBMT) was not reached, and other EM measures—the Brief Visuospatial Memory Test—Revised (BVMT-R) and the Memory Assessments Scales (prose memory) were not significantly improved, and neither was the FAMS (HR-QoL).

Assuming that cognitive training focused on improving new learning via memory-enhancing techniques does not transfer to tasks other than those involved in the intervention and considering that items self-generated by an individual are better remembered than provided information, another method of memory rehabilitation was studied by this group, the self-generation learning program (Self-GEN) [27]. In this program, the therapist teaches patients to discover the benefit of using the self-generation strategy during training, and participants must discuss how the strategies could be used in other tasks. The study involved 35 MS patients and reached primary endpoints (the Contextual Memory Test, an objective learning and memory test, and the Self-Regulation Skills Interview that assesses self-awareness and strategy use). Prospective memory (Memory of Intention Test) and HR-QoL (FAMS) were also improved. However, no significant differences were observed between groups for the CVLT-II, the MFQ, and Actual Reality, which is an ecological assessment of functional performances during an internet-based task. Therefore, the hypothesis that this rehabilitation technique could be transferred to other cognitive functions, notably the executive functions, could not be confirmed.

In a recent pilot study, Self-GEN strategy was associated with two other strategies of memory and learning rehabilitation, space learning (SL), and retrieval practice (RP), in the Strategy-based Training to Enhance Memory (STEM) program [38]. This pilot study, with limited power (20 patients), did not show a significant superiority of the STEM program to the control intervention (non-training program), on the primary endpoint (CVLT-II) and other objective tests of memory (BVMT-R), but it did show some encouraging trends and positive results on HR-QoL (FAMS and Short-Form 36 [SF-36]).

#### 3.1.2. Multiple Domains

Three blinded RCTs were devoted to the individual rehabilitation of cognitive disorders in several domains in MS: two against no intervention and one against a non-specific training program [16,20,34].

The first study compared a 13-week individual NP rehabilitation program in a large sample of patients (99) with no intervention [16,40]. The primary endpoint of this study was the subjective perception of deficits using the Perceived Deficits Questionnaire (PDQ). A greater improvement of this score was observed in the rehabilitated group compared to the control group. Regarding the NP tests, such as the Symbol Digit Modalities Test (SDMT), which was the second primary outcome, and the other tests of the Brief-Repeatable Battery (BRB), no significant improvement was observed, except for the Trial Making Test-A (TMT-A). Note that the intervention’s total duration in this partially negative study was much lower than in the other two individual multidomain studies (780 min versus 3600 and 2250 min).

Although with smaller numbers (43 and 35), the other two studies had significantly better results on the NP tests [20,34]. The first study proposed an individual rehabilitation protocol called MS-Line!, combining written exercises and computer-assisted exercises over a period of 24 weeks compared to no intervention [20]. Although a primary analysis criterion was not specified in the report, the study showed significant results in favor of the treated group for improvement on several NP tests: TMT-A, Spatial Recall Test (SPART-10-36), Letter–Number Sequencing (LNS), Word List Generation (WLG), and the Boston Naming test (BNT). However, the results were negative for some other tests (TMT-B, SDMT, Selective Reminding Test (SRT), and digit span) and HR-QoL scales. Daily cognitive functioning was not studied. No long-term follow-up was done.

The REACTIV study was launched to demonstrate the superiority of a specific CR program (REACTIV) over nonspecific intervention (NSI) for NP assessment, virtual reality cognitive testing, and daily cognitive functioning in MS [34]. It was a single-blind RCT comparing these two interventions in patients with MS selected based on CI at specific tests of IPS, WM, and EF. Both programs included 50 individual sessions administered three times a week for 17 weeks. The specific intervention was tailored to patients’ deficits. The primary endpoint was NP assessment of IPS, attention, EF, and WM. Secondary endpoints included ecological assessment by tasks in a virtual reality environment (Urban Daily Cog^®^) and daily cognitive functioning assessment. More NP scores improved significantly in the active group and several NP scores, alertness and divided attention, and the ecological assessments improved significantly more after specific CR than after NSI. Lastly, SCR improved daily cognitive functioning. Most improvements were maintained 4 months after the end of the intervention. However, HR-Qol was not shown to be improved. The study showed the interest of an individualized and intense intervention including a meta-cognitive approach. It was also the first to show a transfer in ecological tasks. However, the study was underpowered for showing a larger effect on cognitive function. The study was performed in a real-world setting with rehabilitation by speech therapists in city practice.

### 3.2. Group Interventions

Five blinded RCTs have been reported about group intervention, but one of them mixed CT and group intervention [30]. The studies are difficult to compare because of the different rehabilitation programs and the different lengths of rehabilitation.

Among the four group intervention-only studies, the largest study was negative [35]. This study was a single blind study against no intervention and chose the psychological component of the Multiple Impact Scale (MSIS), a HR-QoL scale, as the primary endpoint at 12 months, which was no more improved in the treated group—although a significant small effect was seen at six months. There were no significant differences in NP tests between the two groups. The three other studies showed some positive results [28,29,32]. The first study showed improvements in EM and EF tests, but this study did not identify a primary endpoint [28]. The second study focused solely on the assessment of working memory and showed significant improvement after the group intervention compared to either a group that received relaxation or a group that received no intervention [29]. A double-blind multicenter study compared group rehabilitation to a sham intervention (non-training) [32]. In a fairly large sample of patients selected on the basis of the presence of a cognitive deficit, the main evaluation criterion was EM measured by the Selective Reminding Test (SRT), and a significant difference was observed in the learning curve between the treated group and the control group. WM also improved. However, there was no improvement in the other functions studied (EF, IPS). There was also no positive effect on HR-QoL.

The last study is difficult to interpret because of a mixed intervention combining group management and CT using Lumosity software [30]. Moreover, patients could be included regardless of the presence or absence of CI, and the percentage of patients who were actually cognitively impaired at inclusion is not reported. Overall, the study was negative, the primary endpoint, the Everyday Problems Test—Revised, a self-administered questionnaire, was negative, and among the NP tests, only two were improved: the CVLT and the PASAT. This negative outcome could possibly be due to a significant improvement in the control group treated by a computer game.

### 3.3. Computerized Multidomain Rehabilitation

The largest group of studies devoted to CR in MS is devoted to CT (Table 1 and Table 5).

The RCTs concerning multidomain CR, including attention, IPS, EF, and WM, have been developed in three categories of setting: in institutions, at home with telerehabilitation (online), and at home offline. The duration and number of CT rehabilitation sessions varied from one study to another with sessions lasting 30 to 60 min with a frequency of 2 to 3 per week for a duration of 4 to 12 weeks and a total duration of 300 to 2160 min.

Different software programs were used, the most frequent being RehaCom^®^. Up to 2021, eight studies have been published with this software, and four blinded RCTs were selected for this review [15,22,25,36]. The first study used different modules of the RehaCom^®^ software, delivered in the institution, in the control group than the module evaluated, and this study did not show a clear difference between these modalities [15]. However, the three most recent studies provide fairly consistent evidence for the effectiveness of rehabilitation using RehaCom [22,25,36]. The first study, although of limited power, showed an efficacy of RehaCom^®^ CT at home on the SDMT [22]. The patients were treated by the divided attention, WM, and visuo-spatial memory modules of RehaCom, and the control group received placebo intervention (projection of DVD). The second study compared rehabilitation using RehaCom’s IPS/attention, memory, and EF modules with no treatment in the institution [25]. Although this endpoint was not clearly predefined in the paper, a Time x Group interaction effect was shown by ANOVA on composite z scores for different cognitive domains (verbal EM, attention, fluency, and IPS). The effects were maintained at six months. The same team performed a study in patients with secondary progressive MDS (SPMS) using RehaCom^®^ CT at home compared to sham CT [36]. The CT was performed at home by the patient in the presence of a caregiver and with regular monitoring by a psychologist. This study had the longest total rehabilitation time with the RehaCom^®^ software (1080 min). The primary endpoint (change in Brief International Cognitive Assessment for MS, BICAMS) was achieved with a greater significant improvement of the three BICAMS tests in the treated group as compared to the sham group. HRQoL was also more improved (EQ-5D).

Several studies have looked at other CT programs in MS and are summarized in Table 5.

Three studies used the Posit Science software programs Brain HQ^®^ [16,30] and Insight^®^ [12]. The study by Charvet et al. [16] was the first to show the feasibility of telerehabilitation using a research version of Brain HQ^®^ compared to a computer game. This study showed the feasibility of such a telerehabilitation but with relatively modest results in terms of efficiency (improvement of the composite score without improvement of individual NP scores). A recent study also proposed TR using Brain HQ^®^ but with a lower duration (480 min) [30]. No predefined primary endpoint was mentioned in the report, but several NP tests (EM and EF) were more improved in the treated group than in the control group without any intervention. In the last study, two software programs were used (Insigt from Posit Science and n-back from Brain Twister) [19]. Patients were not selected according to CI. The PASAT (one of the primary outcomes) was the only test more improved in the treated group than in the control group.

Two studies used the Attention processing Training Program (ATP) [17,24]. The study by Amato et al. was the first to show the feasibility of a home-based program [17]. Although this program was developed to improve the different components of attention, only the PASAT test has been improved, highlighting its relatively limited effectiveness. The second study that used APT, in a sample of patients not selected according to the presence of a CI, was negative [24].

The last four studies used different programs (Nintendo, Cogni-Track, Speed of Processing Training (SPT), and Cogmed) [18,21,26,39]. One study compared the telerehabilitation of WM using Cogmed to no intervention and did not show any difference between groups, but some tests improved only in the treated group [39]. A pilot study about SPT in 20 patients did not show any difference between groups on the primary outcomes (NP tests), but IPS improved only in the treated group, and a significant difference between groups was observed for a measure of daily cognitive functioning, the Timed Instrumental Activities of Daily Living Test [26]. The two other studies did not identify a predefined primary endpoint in their report but showed some effects of the Cogni-Track program on some NP tests of EM, WM, IPS, and EF [21] and of Dr. Kawashima’s Brain Training^®^ (Nintendo) on SMT and the Stroop test [18].

## 4. Discussion and Conclusions

The review performed in 2013 by Amato et al. [1] concluded that CR research has methodologic limitations in MS, and although targeted interventions to improve memory and learning show promise, they require further studies. The Cochrane Review published shortly after underlined the low-level evidence for positive effects of neuropsychological rehabilitation in MS [6]. The methodological quality of studies concerning CR in MS has improved significantly compared to the period before 2013. Most of the studies reviewed met most of the quality criteria: RCT, blinded, control group with intervention, defined primary endpoint, assessment of efficacy on neuropsychological tests, but also daily cognitive functioning and HR-QoL.

Altogether, the studies about memory individual CR support the efficacy of m-SMT for improving learning in MS patients. This technique is able to improve verbal episodic memory and HR-QoL. There is some evidence of the efficiency of daily cognitive functioning, but there is no evidence of transfer in other domains. More data are necessary to support other methods of memory individual CR.

The studies about individual multidomain CR are difficult to compare, considering the differences between interventions. The first study used a short CR program (780 min) and did show an improvement in perceived deficits but not in NP scores [16,40]. The second study showed a significant effect on various NP measures but not on HR-QOL [20]. The third study was the only one in this category with an active control group receiving non-specific cognitive training, but was probably underpowered [34]. However, it showed the significant positive superiority of the specific training on several measures of attention but also evidence of improved cognitive functioning and improvement in an ecological task. This study also shows the feasibility of CR in a daily life setting. The results of these two studies support the efficacy of individual specific CR in MS, but they also emphasize the importance of the commitment required from patients who must devote many hours each week, which limits the feasibility of the program for some of them. Overall, most studies of specialized individual rehabilitation have given positive and encouraging results, but this type of rehabilitation requires significant human resources and a significant time investment by patients.

Although group rehabilitation requires fewer resources overall and is easier to implement, the number of studies that have been devoted to it is quite limited, and limited conclusions can be drawn so far. The largest study [35] was negative, one study was positive but without a specified primary endpoint [28], one study was focused only on WM [29], and one combined group CR and CT [30]. The more consistent results are provided by the study of Brissart et al. [32], showing a significant improvement in episodic and working memory. However, no effect on other cognitive domains, daily life cognitive functioning, or HR-QoL was demonstrated.

The large number of studies about CT is probably due to the ease of this technique. The possibility of implementing this CT at home and with telerehabilitation is also very promising. The variety of software used in these studies makes it difficult to compare them. RehaCom has been the most used. Four studies used this software in this review. Three of them showed very encouraging results [22,25,36] on NP scores. Interestingly, the latter [36], performed in SPMS, showed a significant improvement in HR-Qol. All in all, these studies on RehaCom tend to show the effectiveness of this rehabilitation program in MS but leave several important questions unanswered: its use in telerehabilitation, its effectiveness on specific sub-domains according to the cognitive domain most affected, the interest of adding individual rehabilitation sessions including in particular a meta-cognitive approach, its effectiveness on daily cognitive functioning assessed by ecological tests, and its effect on quality of life in RRMS.

The other studies of CT with different programs (APT, PS, SPT) showed various results. As a result of the different software used, it is difficult to draw general conclusions, but some of them demonstrated the feasibility of home-based CR [17,18,21] and of telerehabilitation [23,37,39]. The evidence of the efficacy of telerehabilitation is still limited, but the results encourage further studies in this field. The development of computerized assessment methods for cognitive disorders in MS, which have been the subject of a recent review [42], may help the deployment of telerehabilitation.

Although a demonstration of the effectiveness of multidomain CR, in particular on quality of life and transfer to cognitive domains different from those trained, is lacking, several studies show the feasibility of individual CR in daily life settings and home telerehabilitation using CT. A certain level of efficacy has been established regarding NP outcomes and in several studies on daily cognitive functioning.

## Figures and Tables

**Table 2:** Outcomes.

Batteries	Addenbrooke’s cognitive examination (ACE); Brief-Repeatable Battery (BRB); Wechsler Adult Intelligence Scale (WAIS)
Episodic memory tests	Brief Visuospatial Memory Test—Revised (BVMT-R); California Verbal Learning Test—2nd edition (CVLT-II); Contextual Memory Test (CMT); Greek Verbal Learning Test (GVLT); Hopkins Verbal Learning Test—Revised (HVLT-R); Memory Assessment Scales, Prose Memory (MAS); Memory for Intentions Test (MIST); Memory Functioning Questionnaire (MFQ); Rivermead Behavioral Memory Test (RBMT); Spatial Recall Test (SPART; 10-36); Wechsler Memory Scale-Revised (WMS-R)
Information processing speed tests	Symbol Digit Modalities Test (SDMT)
Attention/Working memory	Letter–Number Sequencing (LNS); Test of Attentional Performance (TAP); Continuous Performance Test (CPT); Paced Auditory Serial Addition Test (PASAT)
Executive functions	Test of Attentional Performance (TAP); Trail-Making Test (TMT); Word List Generation (WLG); Behavior Rating Inventory of Executive Function—Adult (BRIEF-A); Controlled Oral Word Association Test (COWAT); Wisconsin Card-Sorting Test (WCST)
Other	Boston Naming Test (BNT)
Ecological assessment	Actual Reality (AR); Urban DailyCog; Timed Instrumental Activities of Daily Living Test (TIADL)
Quality of Life scales	Functional Assessment of Multiple Sclerosis (FAMS); Mental Health Inventory (MHI); Short Form survey-36 (SF-36); Satisfaction with Life Scale (SWLS); Brief version of the World Health Organization Quality of Life (WHOQoL-BREF); Multiple Sclerosis Impact Scale, psychological (MSIS-psy); Multiple Sclerosis Quality of Life Inventory (MSQLI); EuroQoL five-dimension questionnaire (EQ-5D); MS Quality of Life questionnaire (MSQoL54)
Other self-report scales	Daily Cognitive Activities Questionnaire (DCAQ); Frontal Systems Behavior Scale (FrSBe); Perceived Deficits Questionnaire (PDQ); Self-Regulation Skills Interview (SRSI); Everyday Memory Questionnaire (EMQ); Everyday Problems Test—Revised (EPT-R); Memory Functioning Questionnaire (MFQ); PROMIS (self-reported cognitive function)

References are given in Table 3, Table 4 and Table 5.

**Table 3:** Blinded RCT about individual-specific cognitive rehabilitation.

1st Author	Intervention	Session Duration (Min)	CR Duration (Weeks)	Total Duration (Min)	Control Intervention	Main Positive Results (Primary Outcomes)	LTFU (Mths)	Positive Results (Secondary Outcomes)	Other Positive Results	Main Negative Results (Primary Outcomes Underlined)
Episodic Memory										
Chiaravalloti et al. [14]	m-SMT	45–60 min twice a week	5	450/600	Placebo	CVLT-II, RBMT	6	FAMS		No effect of booster sessions
Goverover et al. [27]	Self-GEN	60 min twice a week	3	360	Placebo	CMT, SRSI	ND	MIST, FAMS		CVLT-II, MFQ, AR
Krch et al. [31]	m-SMT	45 min twice a week	5	450	Placebo	HVLT-R	ND	SWLS		MFQ
Chiaravalloti et al. [33]	m-SMT	45–60 min twice a week	5	450/600	Placebo	CVLT-II	3	FrSBe		RBMT; BVMT-R; MAS, FAMS
Chiaravalloti et al. [38]	STEM	30–45 min twice a week	4	240/360	Placebo	-		SF-36, FAMS, MHI		CVLT
Multidomain										
Rosti-Otajärvi et al. [16,40]	NP intervention	60 min once a week	13	780	No	PDQ	12	TMT-A		WHOQoL-BREF, SDMT
Gich et al. [20]	MS-Line!	75 min twice a week	24	3600	No	No specified primary outcome	ND		SPART, LNS, WLG, BNT, TMT-A	digit span, SDMT, SRT, TMT-B
Lamargue et al. [34]	REACTIV	45 min, 3 times a week		2250	Non-specific training	TAP (Alertness, Divided Attention)	4	CVLT, Rey figure copy; Urban Daily-Cog, DCAQ		SDMT *, n-Back, TMT

CR: cognitive rehabilitation; Min: minutes; LTFU: positive results at long term follow-up; ND: not done; Mths: months; *: test improved in both groups. Rehabilitation programs: m-SMT: modified-story memory technique; Self-Gen: self-generation learning program; STEM: Strategy-based Training to Enhance Memory; MS-Line!: NP intervention using written, manipulative, and computer-based materials; REACTIV: NP intervention using written and computer-based materials. Outcomes: AR: Actual Reality; BNT: Boston Naming Test; BVMT-R: Brief Visuospatial Memory Test—Revised; CMT: Contextual Memory Test; CVLT-II: California Verbal Learning Test—2nd edition; DCAQ: Daily Cognitive Activities Questionnaire. FAMS: Functional Assessment of Multiple Sclerosis; FrSBe: Frontal Systems Behavior Scale; HVLT-R: Hopkins Verbal Learning Test—Revised; MAS: Memory Assessment Scales, Prose Memory; MIST: Memory for Intentions Test; MFQ: Memory Functioning Questionnaire; MHI: Mental Health Inventory; LNS: Letter–Number Sequencing; PDQ: Perceived Deficits Questionnaire; RBMT: Rivermead Behavioral Memory Test; SDMT: Symbol Digit Modalities Test; SF-36: Short Form survey-36; SPART: Spatial Recall Test (10–36); SRSI: Self-Regulation Skills Interview; SWLS: Satisfaction with Life Scale; TAP: Test of Attentional Performance; TMT: Trail-Making Test; Urban DailyCog: Ecological test in a virtual realty environment; WHOQoL-BREF = Brief version of the World Health Organization Quality of Life; WLG: Word List Generation. Underlined outcomes are primary outcomes.

**Table 4:** Group Interventions blinded RCT.

1st Author	Intervention	Session Duration (Min)	CR Duration (Weeks)	Total Duration (Min)	Control Intervention	Main Positive Results (Primary Outcomes)	LTFU (Mths)	Positive Results (Secondary Outcomes)	Other Positive Results	Main Negative Results (Primary Outcomes Underlined)
Multidomain										
Mani et al. [28]	Group + homework	120 min twice a week	4	960	No training (sham intervention)	No specified primary outcome	4		ACE (memory), BRIEF-A, MFQ WCST, WMS-R	CPT (attention)
Moussavi et al. [29]	Group (4 persons) + homework	60 min once a week	8	480	Relaxation and no intervention	Working Memory (WMS-R)	4	-		
Stuifbergen et al. [30]	Group + CT at home (Lumosity)	120 min once a week + 45 min, 3 times a week	8	Variable Up to 2040	No intervention (games)	-	6	CVLT, PASAT		EPT-R, BVMT-R, COWAT, SDMT, PROMIS
Brissart et al. [32]	Group	120 min twice a month	24	1560	No training (placebo intervention)	SRT (learning index)	ND	Digit span backward, TAP (WM)		Flexibility (TAP), verbal fluency, IPS (WAIS digit-symbol), SPART; MSQLI
Lincoln et al. [35]	Group (4–6 persons)	90 min each week	10	900	No intervention	-	12	EMQ MSIS (6Mths),		MSIS-psy (12Mths), BRB tests

CR: cognitive rehabilitation; Min: minutes; LTFU: positive results at long term follow-up; ND: not done; NP: neuropsychological; W: week; Mths: months; IPS: information processing speed; WM: working memory. Outcomes: ACE: Addenbrooke’s cognitive examination; BRB: Brief-Repeatable Battery; BRIEF-A: Behavior rating inventory of executive function-adult; BVMT-R: Brief Visuospatial Memory Test—Revised; COWAT: Controlled Oral Word Association Test; CPT: Continuous Performance Test; CVLT-II: California Verbal Learning Test—2nd edition; EMQ: Everyday Memory Questionnaire EPT-R: Everyday Problems Test—Revised; MFQ: Memory Functioning Questionnaire; MSIS-psy: Multiple Sclerosis Impact Scale, psychological; MSQLI: Multiple Sclerosis Quality of Life Inventory; PASAT: Paced Auditory Serial Addition Test; PROMIS: self-reported cognitive function; SDMT: Symbol Digit Modalities Test; SPART: Spatial Recall Test (10–36); SRT: Selective Reminding Test; TAP: Test of Attentional Performance; WAIS: Wechsler Adult Intelligence Scale; WCST: Wisconsin Card-Sorting Test; WMS-R: Wechsler Memory Scale—Revised. Underlined outcomes are primary outcomes.

**Table 5:** Blinded RCT of CT in MS.

1st Author	Year	Intervention (Software)	Session Duration (Min)	CR Duration (Weeks)	Total Duration (Min)	Control Intervention	Main Positive Results (Primary Outcomes)	LTFU (Mths)	Positive Results (Secondary Outcomes)	Other Positive Results	Main Negative Results (Primary Outcomes Underlined)
Cerasa et al. [15]	2013	RehaCom	60 min twice a week	6	720	Placebo training	no specified primary outcome	ND		ST	BRB tests, TMT
Amato et al. [17]	2014	APT	60 min twice a week	12	1440	Placebo training	no specified primary outcome	6		PASAT	SDMT *
De Giglio et al. [18]	2015	DKBT	30 min 3 times a week	8	720	No intervention	no specified primary outcome	ND		ST, SDMT, some MSQoL54 subscales	PASAT
Hancock et al. [19]	2015	PS Insight and Brain Twister n-back	30 min 6 times a week	6	1080	Sham training	PASAT	ND			SDMT, LNS, Digit backward
Pedullà et al. [21]	2016	Cogni-Track	30 min 5 times a week	8	1200	Non-adaptative training	no specified primary outcome	6		SRT, SDMT, PASAT, WLG	SPART, WCST
Campbell et al. [22]	2016	RehaCom	45 min 3 times a week	6	810	Placebo training	SDMT	3			BVMT, CVLT, FAMS, EQ-5D
Charvet et al. [23]	2017	Brain HQ (PS)	60 min 5 times a week	12	1800	Placebo training	Composite NP score	ND			
Grasso et al. [24]	2017	APT	60 min 3 times a week	12	2160	No intervention	no specified primary outcome	6	**		
Messinis et al. [25]	2017	RehaCom	60 min twice a week	10	1200	No intervention	no specified primary outcome	6		Verbal EM, attention, verbal fluency, IPS z scores	
Chiaravalloti et al. [26]	2018	SPT	30 min twice a week	5	300	No intervention		ND	TIADL		WAIS digit symbol *
Messinis et al. [36]	2020	RehaCom	45 min 3 times a week	8	1080	Placebo training	SDMT, GVLT, BVMT	ND	EQ-5D		
Vilou et al. [37]	2020	Brain HQ (PS)	40 min twice a week	6	480	No intervention	no specified primary outcome	ND		GVLT, BVMT, TMT-A, ST	SDMT
Blair et al. [39]	2021	Cogmed	30–45 min 5 times a week	5	750–1125	No intervention		6			PASAT *, SDMT *, ST *

CR: cognitive rehabilitation; Min: minutes; LTFU: positive results at long-term follow-up; ND: not done; Mths: months; EM: episodic memory; IPS: information processing speed; *: test improved in both groups; ** no difference between groups. Rehabilitation programs: ATP: Attention Processing Training program; DKBT: Dr. Kawashima’s Brain Training^®^ (Nintendo); PS: Posit Science; SPT: Speed of Processing Training. Outcomes: BRB: Brief-Repeatable Battery; BVMT-R: Brief Visuospatial Memory Test—Revised; CVLT-II: California Verbal Learning Test—2nd edition; EQ-5D: EuroQoL five-dimension questionnaire; FAMS: Functional Assessment of Multiple Sclerosis; GVLT: Greek Verbal Learning Test; LNS: Letter–Number Sequencing; MSQoL54: MS Quality of Life questionnaire; PASAT: Paced Auditory Serial Addition Test; SDMT: Symbol Digit Modalities Test; SPART: Spatial Recall Test (10–36); SRT: Selective Reminding Test; ST: Stroop test; TIADL: Timed Instrumental Activities of Daily Living Test; TMT: Trail-Making Test. WAIS: Wechsler Adult Intelligence Scale; WCST: Wisconsin Card-Sorting Test; WLG: Word List Generation. Underlined outcomes are primary outcomes.
